# The impact of the COVID‐19 pandemic on the care of pulmonary hypertension patients outside the Hubei province in China

**DOI:** 10.1002/pul2.12130

**Published:** 2022-07-01

**Authors:** Yuqin Chen, Bihua Zhong, Qian Jiang, Yilin Chen, Wenjun He, Ning Lai, Dansha Zhou, Jiahao He, Yiting Yao, Yi Shen, Juan Li, Jianuo Yang, Zhe Zhang, Ran Ma, Jian Wang, Chunli Liu

**Affiliations:** ^1^ State Key Laboratory of Respiratory Disease, National Center for Respiratory Medicine, National Clinical Research Center for Respiratory Disease, Guangdong Key Laboratory of Vascular Disease, Guangdong‐Hong Kong‐Macao Joint Laboratory of Respiratory Infectious Disease, Guangzhou Institute of Respiratory Health, The First Affiliated Hospital of Guangzhou Medical University Guangzhou China; ^2^ Department of Respiratory and Critical Care Medicine The Second People's Hospital of Foshan (Affiliated Foshan Hospital of Southern Medical University) Foshan Guangdong China; ^3^ Department of Pulmonary Medicine, PHEniX Laboratory Amsterdam UMC location Vrije Universiteit Amsterdam Amsterdam The Netherlands; ^4^ Amsterdam Cardiovascular Sciences, Pulmonary Hypertension and Thrombosis Amsterdam The Netherlands

**Keywords:** COVID‐19, medical treatment, pulmonary hypertension, questionnaire

## Abstract

The Coronavirus disease 2019 (COVID‐19) pandemic has severely affected the lives of people around the world, especially some patients with severe chronic diseases. This study aims to evaluate the impact of the COVID‐19 outbreak from December 2019 to April 2020 on treating patients with PH. A questionnaire regarding the medical condition of PH patients during the COVID‐19 pandemic was designed by PH diagnostic experts in The First Affiliated Hospital of Guangzhou Medical University, China Respiratory Center. One hundred and fifty‐six subjects with PH from non‐Hubei regions in China were invited to participate in this survey online. 63.4% (*n* = 99) of them had difficulty seeing a doctor, and the main reason was fear of contracting severe acute respiratory syndrome coronavirus 2 (SARS‐CoV‐2) in the hospital. Medical treatment was affected in 25% (*n* = 39) of patients, and who lived in rural areas, and discontinued medical therapy for financial reasons were at a higher risk of medical treatment being affected. Patients who reduced nutrition, and had difficulty seeing a doctor were more likely to get deteriorated. During the epidemic, the hospitalization rate of PH patients was 33.33%. Patients with aggravated PH had a high risk of hospitalization (odds ratio [OR] = 2.844), while patients who visited a doctor during the epidemic reduced the risk of hospitalization (OR = 0.33). In conclusion, during the COVID‐19 pandemic, PH patients had difficulty seeing a doctor, and their medical treatment was affected, even worsened, and increased the risk of hospitalization.

## INTRODUCTION

Pulmonary hypertension (PH) is a syndrome characterized by progressive elevation of pulmonary vascular pressure and resistance, leading to right heart failure or even death.^1^ PH is defined by a mean pulmonary artery pressure >20 mmHg at rest, measured by right heart catheterization.^2^ It is a chronic disease that requires maintenance therapy. According to statistics, the global prevalence of PH is about 1%, with 10% aged 65 years.^3^ About 80% of patients from developing countries have their lives affected.^3^ The median survival in idiopathic pulmonary arterial hypertension (IPAH) was 2.8 years, and in the first, third, and fifth year survival rates in untreated IPAH patients were 68%, 48%, and 34%, respectively.^4^ With the development of targeted therapy, the prognosis of IPAH has improved significantly. The US REVEAL registry, which enrolled data from 2716 patients, showed that the 1‐, 3‐, 5‐, and 7‐year survival rates of PAH patients were 85%, 68%, 57%, and 49%, respectively.^5^, ^6^ Pharmacological therapy is the key part of treatment for most PH patients. In China, most chronic‐ill patients must be prescribed medication at the hospital every month.

Coronavirus disease 2019 (COVID‐19), caused by severe acute respiratory syndrome coronavirus 2 (SARS‐CoV‐2), was first identified in Wuhan, China, in December 2019.^7^ On March 11, 2020, the outbreak of SARS‐CoV‐2 was declared a pandemic by the World Health Organization (WHO) due to the high transmissibility of the virus and the rapid increase in the number of cases. A pandemic triggered by SARS‐CoV‐2 has caused more than 4.2 million people worldwide to become infected and killed more than 280,000 people, according to Johns Hopkins University, until April 2020. From January 23 to April 8, 2020, Wuhan was locked down for 76 days due to the COVID‐19 pandemic, which is an important event point in the full outbreak of the SARS‐CoV‐2 and the escalation of prevention and control measures in China.^8^, ^9^ However, the impact of the pandemic on people's livelihood and public health care goes well beyond 76 days.^8^, ^9^ During the lockdown period, hospitals suspended outpatient services, and the city imposed traffic control. Patients' economic situation deteriorated, and fear of the SARS‐CoV‐2 led to a reluctance to seek medical treatment. All of which made it difficult for patients with PH to seek treatment.^10^

PH is a serious chronic disease that requires long‐term, regulated treatment.^11^ With the emergency closure and traffic control of Wuhan, Hubei province, it was inevitable that the medical treatment of patients would be affected. Zhou et al.^12^ have confirmed this. However, was the medication for PH patients also affected in areas outside Hubei where the epidemic situation is relatively mild? To investigate this issue, we designed a questionnaire to collect information from PH patients in the non‐Hubei regions of China to assess the impact of COVID‐19 on them, aiming to provide a basis for developing more rational disease management measures for these patients during the pandemic.

## METHODS

### Study design

The questionnaire regarding the medical condition of PH patients during the COVID‐19 pandemic was designed by PH diagnostic experts in The First Affiliated Hospital of Guangzhou Medical University, National Respiratory Center in China. The whole questionnaire was composed of three parts, and 49 questions were designed. The first part is the patient's basic information, the second part is about the diagnosis of PH and the condition of medication before the pandemic, and the third part is the impact of the pandemic on patients' medical treatment and condition. And then, we conducted an online survey to visit patients with PH by WeChat or telephone at seven different pulmonary vascular centers in China to collect basic demographic and disease information. Questionnaires were collected between April 18 and May 7, 2020.

A total of 208 patients participated, verbal permission was obtained from 161 patients, and 161 questionnaires were collected. Inclusion criteria for the study were: (1) patients diagnosed with pulmonary hypertension for at least 3 months; and (2) patients or their guardians who could accurately understand and answer the questionnaire. We excluded: (1) patients who lived in Hubei during the epidemic, (2) subjects with incomplete information, and (3) patients who were not able to complete the questionnaire.

### Statistical analysis

Categorical data described data distribution with frequencies and percentages, and *χ* ^*2*^ tests were used for between‐group comparisons. For nonnormally distributed continuous data, the median and interquartile range were used to describe the data distribution, while the rank sum test was used to compare groups. For normal data, mean and standard deviation were used to describe the data distribution, the Student's *t*‐test was used to compare two groups, and analysis of variance was used to compare three or more groups. The univariate analysis's variables with *p* < 0.05 were subjected to binary logistic regression analysis. *p*< 0.05 was considered statistically significant. All statistical analyses were performed with SPSS 25.0 (IBM Corp.).

## RESULTS

### Participants

Two hundred and eight patients were invited to participate in this online survey who from seven non‐Hubei provinces between April 18 and May 7, 2020. Until May 7, 161 (77.40%) individuals completed and returned the questionnaires. Among them, 156 (96.89%) patients met the entry criteria and were included in the statistics. The characteristics of patients with PH are summarized in Table 1. The patients enrolled in this study were divided into five subgroups (% of total patients): Ia: Idiopathic pulmonary arterial hypertension (18.59%); Ib: PH due to congenital heart disease (9.62%); Ic: PH due to connective tissue disease (7.05%); III: PH due to lung disease and/or hypoxia (40.38%); IV: chronic thromboembolic PH (12.82%), and PH due to miscellaneous causes (11.54%). The mean age at the study time was 52.93 ± 17.56 years, with a 1:0.88 female:male ratio. The ratio of urban to rural population was about 1.33:1. Most respondents have low education, and 85.26% (*n* = 133) of the patients did not exceed high school education. 49.36% (*n* = 77) of households had an annual income of less than 50,000 yuan (according to the US official poverty line in 2018, the minimum standard family annual income is 12,043 dollars, equivalent to 81,196.31 yuan). 73.72% (*n* = 115) patients had an annual personal income of less than 30,000 yuan, while 10.26% (*n* = 16) had the national low‐income population guarantee. However, 32.05% (*n* = 50) of PH patients have an annual medical expenditure of not less than 50,000 yuan. None of the patients were infected with the novel coronavirus.

**Table 1:** Demographics of the patients with PH

Characteristic	Patient
Age, year	52.93 ± 17.56
Female, *n* (%)	83 (53.20)
Rural residents, *n* (%)	67 (42.95)
Education level, *n* (%)	
Primary school or below	46 (29.49)
Middle and high school	87 (55.77)
College and higher	23 (14.74)
Income, *n* (%)	
Family's income <50,000 yuan/year	77 (49.36)
Personal income <30,000 yuan/year	115 (73.72)
Medical expenses, *n* (%)	
<50,000 yuan/year	106 (67.95)
≥50,000 yuan/year	50 (32.05)
PH group, *n* (%)	
IPAH	29 (18.59)
CHD‐PH	15 (9.62)
CTD‐PH	11 (7.05)
Respiratory disease‐PH	63 (40.38)
CTEPH	20 (12.82)
Miscellaneous causes	18 (11.54)
Use of anti‐PH medication, *n* (%)	
Sildenafil	38 (24.36)
Tadalafil	36 (23.08)
Ambrisentan	35 (22.44)
Macitentan	22 (14.10)
Riociguat	20 (12.82)
Bosentan	20 (12.82)
Selexipag	15 (9.62)
Beraprost	15 (9.62)
Treprostinil	10 (6.41)
Diuretics	48 (30.77)

Abbreviations: CHD‐PH, pulmonary hypertension due to congenital heart disease; CTD‐PH, pulmonary hypertension due to connective tissue disease; CTEPH, chronic thrombotic embolism pulmonary hypertension; IPAH, Idiopathic pulmonary hypertension; PH, pulmonary hypertension.

### The difficulty of seeing a doctor

**Figure 1:**
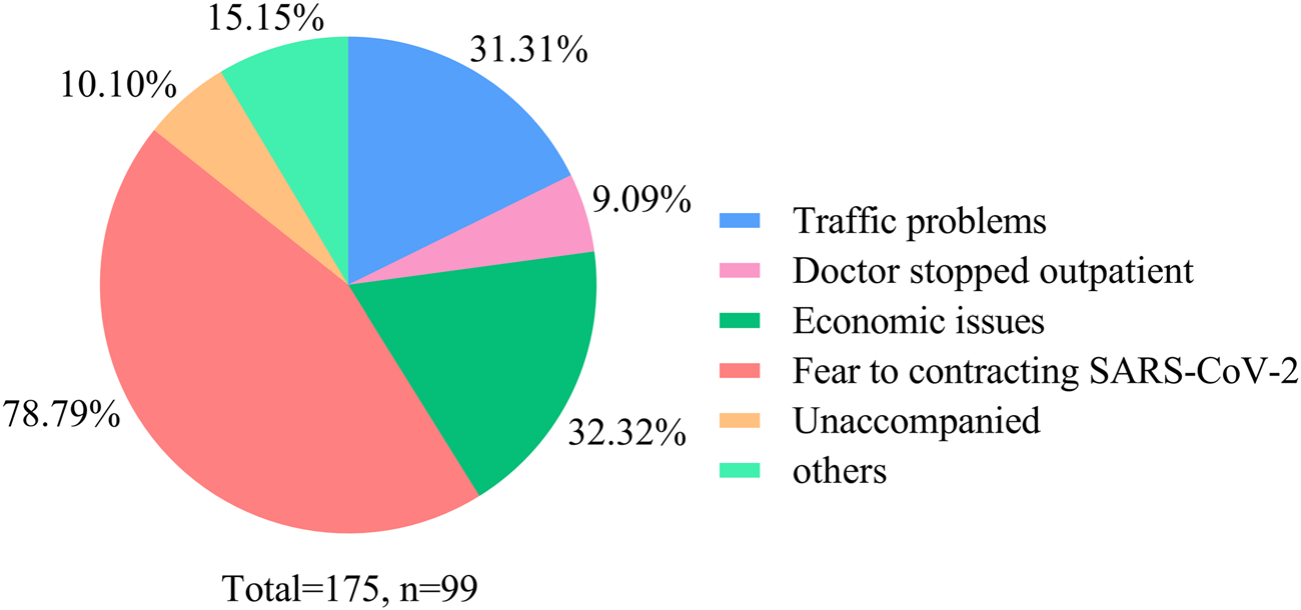
Subjective reasons for difficulty seeing a doctor in patients with pulmonary hypertension. Different colors of the pie show different reasons, fear of contracting SARS‐CoV‐2 was the main reason, followed by economic issues and traffic problems.

Before the COVID‐19 pandemic, 62.18% (*n* = 97) have come to the hospital on time for follow‐up visits within 3 months. During the pandemic, only 55.13% (*n* = 86) of PH patients visited the hospital, and most of them were consulted by their families. 63.46% (*n* = 99) had difficulty seeing a doctor. The main reason was fear of contracting SARS‐CoV‐2 (78.79%), followed by economic issues (32.32%), traffic problems (31.31%), unaccompanied (10.10%), doctors stopped outpatient (9.09%), and other reasons (15.15%), such as feeling unwell wearing a mask when going out, and not taking medicines regularly for fear of being blamed by the doctors (Figure 1). However, only 30.77% (*n* = 48) tried using multimedia to contact their doctors. New media, such as WeChat, was their most common way (79.17%). 72.9% of patients felt that their problems could be solved by contacting doctors through multimedia, and 27.08% of them felt that their problems could not be solved, while the main reasons were that they felt doctors could not appropriately judge their condition (32.69%), with reasons that they could not conduct tests (28.85%) and obtain a prescription (26.92%), and had communication barriers (19.23%). Among the patients who used multimedia to communicate with doctors, 77.08% considered the effect was not as good as the on‐site outpatient, while 22.92% believed that the results were the same as the on‐site outpatient.

### Medical treatment was affected

**Figure 2:**
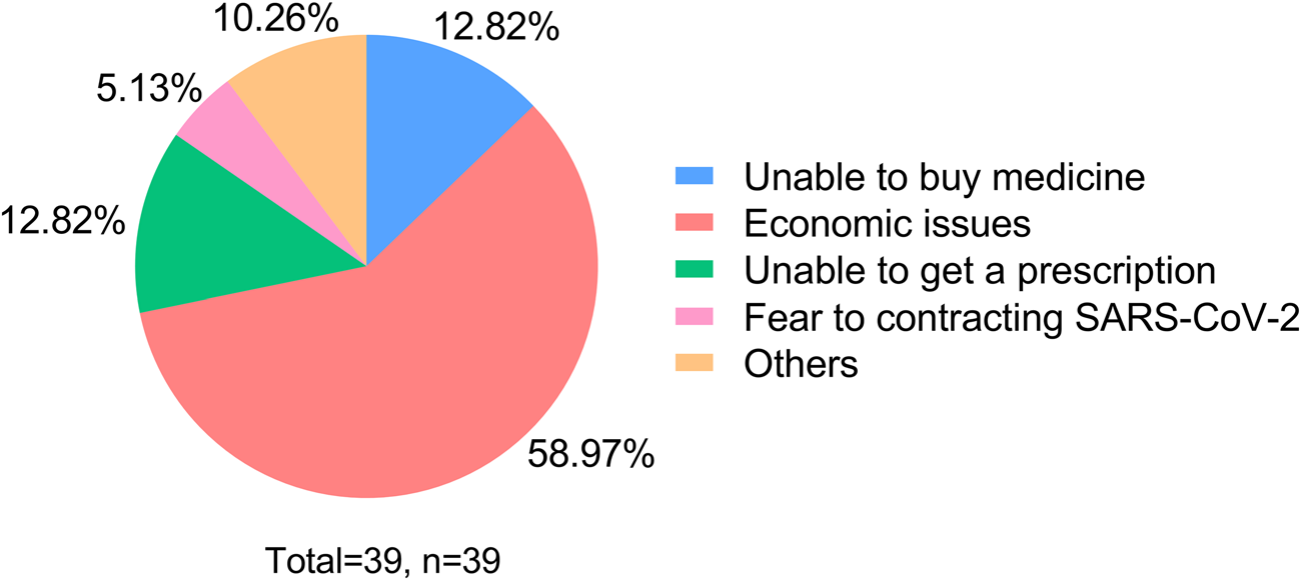
The subjective reasons for medication treatment were affected. Different colors of the pie represent different reasons, economic problem was the main reason, followed by unable to buy medicine and get a prescription.

**Figure 3:**
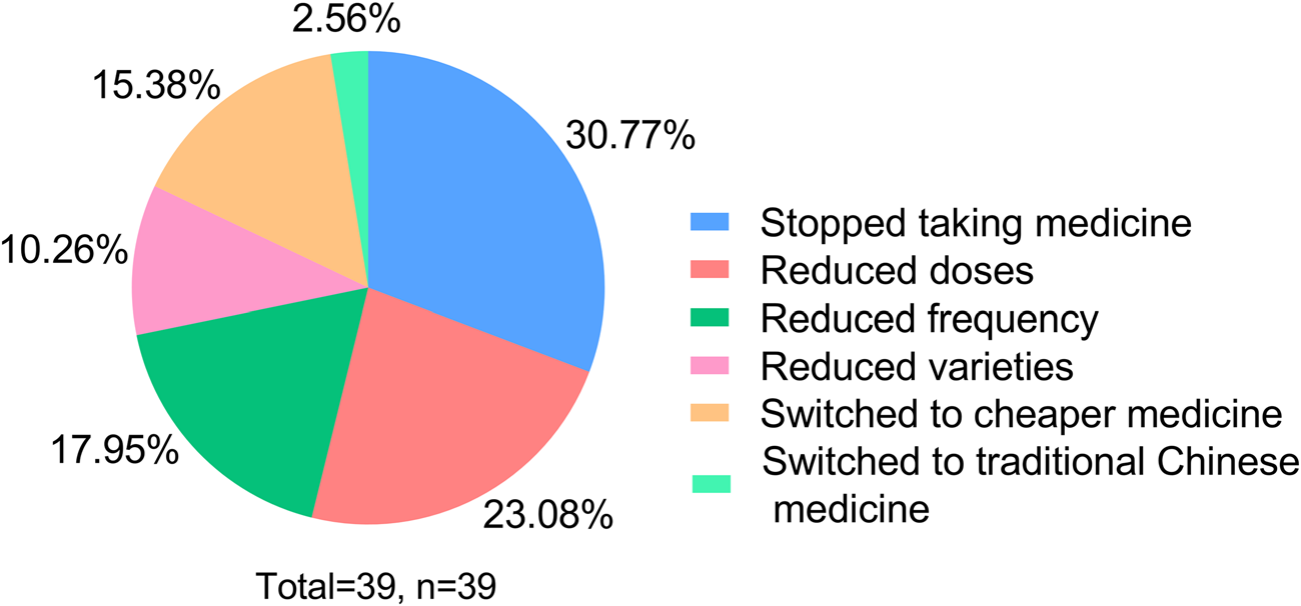
How the patients surveyed responded to the effects of drug therapy. The different colors in the pie express different ways, the percentage shows the proportion, and most people have stopped or reduced their drugs.

**Figure 4:**
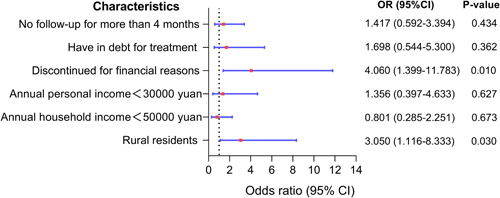
Multivariate analysis of medical treatment affected during COVID‐19 pandemic. The ordinate represents different factors, the abscissa represents the odds ratio (OR) and the 95% confidence interval (CI) (the red dot represents the OR, and the blue horizontal line shows the size of the CI), and the right side shows the specific OR value, 95% CI and *p* value.

Sildenafil, Tadalafil, and Ambrisentan were the most commonly used target therapy medications. Specifically, Sildenafil and Tadalafil were preferred in 24.36% (*n* = 38) and 23.08% (*n* = 36) patients. During the pandemic, 25% (*n* = 39) of patients' medication treatment was affected. The subjective reasons were economic issues (58.97%), unable to buy medicine (12.82%), unable to get a prescription (12.82%), fear of going out and contracting SARS‐CoV‐2 (5.13%), and others (10.26%), such as the onset of other diseases, did not register and did not dare to take medicines by themselves. (Figure 2). During that time, they mainly got their medicine from hospitals (59.62%), followed by physical pharmacies (18.59%), and online pharmacies (10.26%). As seen in Figure 3, in the face of the impact, 30.77% of the patients stopped taking medicine, 23.08% reduced doses, 17.95% reduced frequency, 15.38% switched to cheaper medicine, 10.26% reduced the varieties of medicine, and 2.56% switched to traditional Chinese medicine. According to univariate analysis, living area (*χ* ^*2*^ = 17.66, *p*<0.001), annual family income (*χ* ^*2*^ = 6.23, *p* = 0.013), personal annual income (*χ* ^*2*^ = 4.86, *p* = 0.027), discontinued medical therapy for financial reasons (*χ* ^*2*^ = 28.82, *p*< 0.001), having been in debt due to medical treatment (*χ* ^*2*^ = 20.94, *p*< 0.001), and having not been to the hospital for a follow‐up visit for more than 4 months (*χ* ^*2*^ = 6.03, *p* = 0.014) were considered to be the influencing factors of medical treatment. Incorporating the above factors *p* < 0.05 into the binary logistic regression analysis (Figure 4), it was found that patients lived in rural areas (odds ratio [OR] = 3.05, 95% confidence interval [CI]: 1.116–8.333, *p* = 0.03) and discontinuous medication for financial reasons (OR = 4.06, 95% CI: 1.399–11.783, *p* = 0.01) were at a high risk of medical treatment being affected.

### Pulmonary hypertension progression and had a high risk of hospitalization

**Figure 5:**
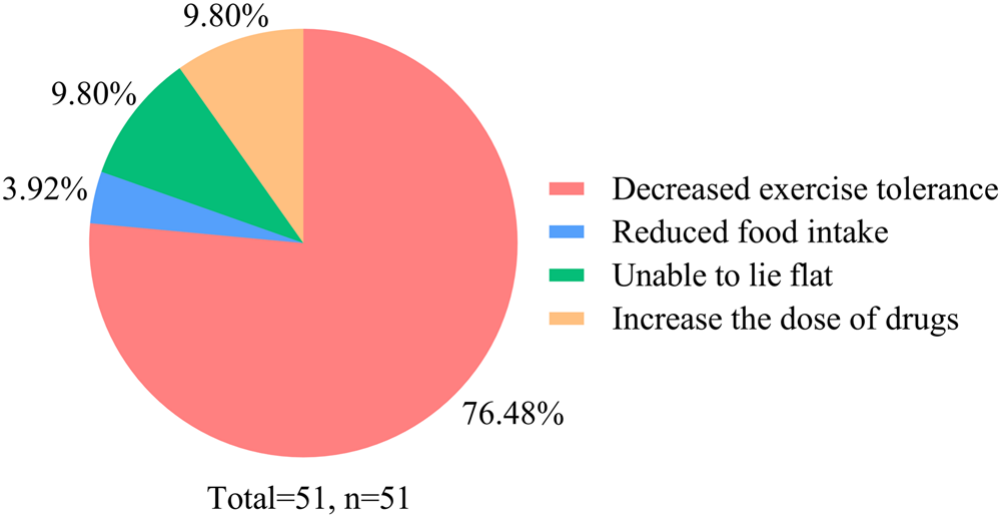
Clinical manifestations of patient deterioration. Different colors are used to show different clinical manifestations, and most of them show decreased activity tolerance.

**Figure 6:**
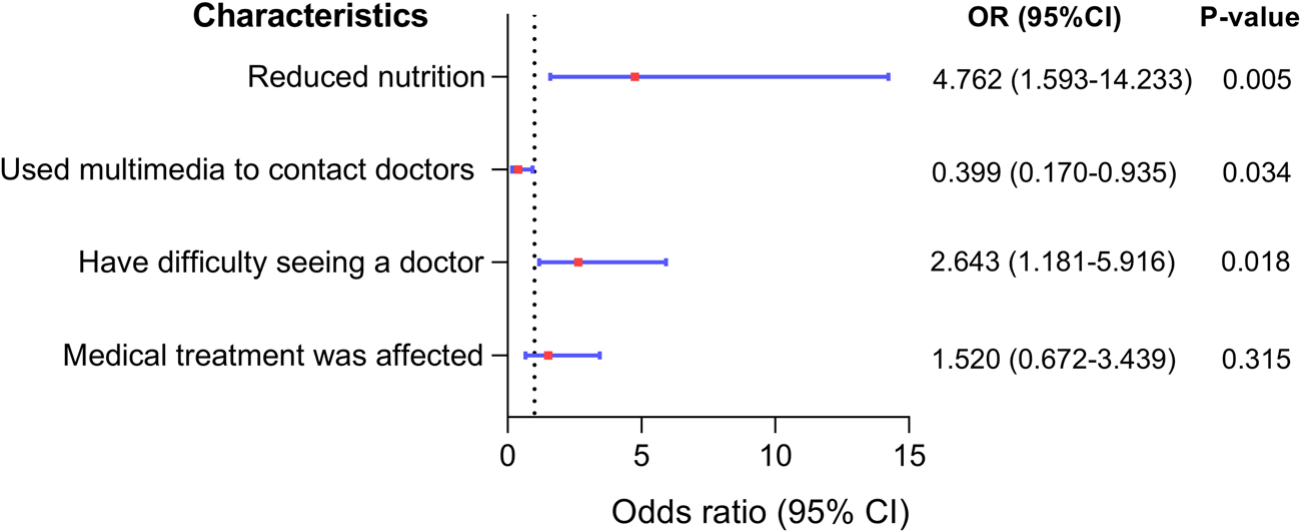
Multivariate analysis of disease progression during COVID‐19 pandemic in PH patients. The ordinate represents different factors, the abscissa represents the odds ratio (OR) and the 95% confidence interval (CI) (the red dot represents the OR, and the blue horizontal line shows the size of the CI), and the right side shows the specific OR value, 95% CI and *p* value.

In our survey, 32.69% (*n* = 51) felt that their PH had deteriorated, 29.49% (*n* = 46) felt similar to before, while 37.82% (*n* = 59) felt improved. As shown in Figure 5, 76.47% of the exacerbated patients had decreased exercise tolerance, 9.80% could not lie flat, 9.80% needed to increase the dose of drugs, and 3.92% reduced food intake. Univariate analysis revealed that having difficulty in seeking a doctor (*χ* ^*2*^ = 5.53, *p* = 0.019), contacting doctors by multimedia (*χ* ^*2*^ = 4.43, *p* = 0.035), nutritional status (*χ* ^*2*^ = 10.67, *p* = 0.001), and medical treatment being affected (*χ* ^*2*^ = 6.42, *p* = 0.011) were the influencing factors for the progression of PH. To exclude confounding factors, we performed a binary logistic analysis and included the model according to the standard of *p* < 0.05 (Figure 6) and found that reduced nutrition (OR = 4.672, 95% CI: 1.593–14.233, *p* = 0.005), having difficulty in seeing a doctor (OR = 2.643, 95% CI: 1.181–5.916, *p* = 0.034), and contacting doctors by multimedia (OR = 0.399, 95% CI: 0.170–0.935, *p* = 0.0182) were the independent influencing factors. Furthermore, the first two were risk factors, while contacting doctors via multimedia was a protective factor. During the epidemic, the hospitalization rate of PH patients was 33.33%. After adjustment for potential confounders (gender, age, marriage, living area, economic status, etc.), the patients with aggravated PH had a significantly high risk of hospitalization (OR = 2.844, 95% CI: 1.137–7.133, *p* = 0.025), while patients who have visited doctors during the epidemic reduced the risk of hospitalization (OR = 0.33, 95% CI: 0.005–0.212, *p* < 0.001).

## DISCUSSION

COVID‐19 is an outbreak of a rapidly spreading infectious disease caused by SARS‐CoV‐2. In late December 2019, several local health institutions in Wuhan reported a group of patients with pneumonia of unknown cause. On December 31, 2019, the Chinese Center for Disease Control and Prevention (CDC) dispatched a rapid response team to accompany health authorities in Wuhan, Hubei province, to conduct epidemiological and pathogenic investigations.^7^ According to international CDC data (https://www.cdc.gov/), as of March 2021, the global COVID‐19 pandemic spread to all continents, with more than 146.8 million confirmed cases and over 3 million deaths. The COVID‐19 outbreak has caused major socioeconomic problems, overwhelmed health services worldwide, and severely affected the quality of life.^13^, ^14^ Some articles have reported that it can also impact mental health, leading to anxiety and depression.^15^, ^16^, ^17^ SARS‐CoV‐2 is highly contagious and is primarily transmitted through breath aerosols, direct contact, and fecal‐oral.^18^ Specifically, patients with chronic diseases might have both high‐risk COVID‐19 and uncontrolled existing diseases.^19^ PH was associated with higher in‐hospital mortality or intensive care unit admission in a 2020 single‐center clinical study.^20^ Patients with PH require ongoing medical care similar to other chronic diseases. Any drug interruption may lead to clinical deterioration and death. In Hubei province, chronic disease patients were affected when Wuhan was sealed off.^12^ However, patients' living condition during the COVID‐19 outbreak was unknown in non‐Hubei regions. Our small national survey has brought insight into this group.

During the pandemic, the normal outpatient services of many hospitals were affected. Especially most chronic disease and specialist outpatient services have been closed. Our small‐scale study investigated 156 patients and found that 63.46% had difficulty seeing a doctor then. Furthermore, 55.13% of patients did not return to the hospital during the pandemic. The main reasons were the fear of contracting SARS‐CoV‐2 in hospitals, economic issues, and traffic problems. As mentioned earlier, the COVID‐19 outbreak has caused major socioeconomic problems and severely affected mental health.^13^, ^14^ Multimedia was used more and more widely in health management. Especially during the COVID‐19 pandemic in China, to reduce the concentration of patients in hospitals and decrease the risk of contracting SARS‐CoV‐2, many hospitals have gradually used online consultation platforms. However, only 30.77% of patients tried contacting their doctor in multimedia format in our study. Nearly 80% of those had a worse experience than the on‐site outpatient. The main reasons were that they felt doctors could not appropriately judge their condition, they could not conduct tests and obtain a prescription, and having communication barriers.

PH is a chronic disease that requires maintenance medication. Although the medical burden is heavy, most patients have high medication compliance.^21^ In our survey, all patients used medications before the pandemic. Moreover, 25% of patients' medical treatment was affected. Most patients experienced interruptions in medical therapy or changed their treatment plan without consulting their doctor; some even switched to traditional Chinese medicine. Our results show that medical treatment was much less affected than the small‐scale survey in Hubei, which found that 70% (*n* = 100) of participants implied a shortage in medications, and 24.2% (*n* = 29) of them stopped medical treatments when the COVID‐19 outbreak in Hubei.^12^ This may be due to the fact that there was no city blockade and traffic controls in non‐Hubei areas, and the number of SARS‐CoV‐2 infections was not so big.

Regarding the reasons for the impact of medical treatment, more than half of the patients (58.97%) chose economic pressure. We also found that patients who lived in rural areas (*p* < 0.001), with family income <50,000 yuan/year (*p* = 0.013) and personal income <30,000 yuan/year (*p* = 0.027), discontinued medical therapy for financial reasons (*p*< 0.001), had been in debt due to medical treatment (*p*< 0.001), and missed hospital visits for more than 4 months (*p* = 0.014) were vulnerable to medical treatment during the COVID‐19 pandemic. During the pandemic, economically disadvantaged and mobility‐impaired patients were vulnerable to socioeconomic hits. However, after conducting a multivariate analysis to eliminate confounding factors, only patients who lived in rural areas (OR = 3.050, *p* = 0.030) and discontinued medical therapy for financial reasons (OR = 4.060, *p* = 0.010) had a high risk of medical treatment being affected.

Although PH is a chronic disease that requires long‐term standardized treatment.^11^ Patients without standardized treatment are at high risk of deterioration and even death. COVID‐19 has disrupted people's lives and also affected the medical treatment of many patients with chronic diseases, which may impact their condition. Our research studied the progress of PH and found that 32.69% (*n* = 51) felt that their PH had deteriorated, mainly manifesting as decreased activity tolerance, the most common clinical symptom of PH. Patients with difficulty in seeing a doctor (*p* = 0.019), poor nutritional status (*p* = 0.001), and medical treatment affected (*p* = 0.011) were more likely to worsen, while patients using multimedia to contact doctors (*p* = 0.035) were less likely to worsen. The patient's condition worsened due to the inability to seek medical attention on time. Malnourished patients may suffer from loss of appetite due to pandemic‐induced anxiety and depression, which may lead to worsening pH, or worsening health status leading to malnutrition, both of which interact. PH was likely to worsen by stopping or reducing medication in those affected by medical treatment. In addition, the patients with aggravated PH also led to high‐risk hospitalization, while patients who visited doctors during the epidemic reduced the risk.

However, our survey has some shortcomings. Because it could not communicate face‐to‐face with patients and guide them to fill in correctly during the pandemic, this may lead to incorrect and comprehensive information. In addition, some elderly people ask family members to help fill in, which may cause the information to be biased. On the other hand, although multiple centers distribute our questionnaire, the sample size is still not large enough. More patients and longer visits should be considered to understand the progress of their disease and the development of various factors.

## CONCLUSIONS

As expected, our research found that during the COVID‐19 pandemic, most PH patients from non‐Hubei areas also had difficulty seeing a doctor. The epidemic has affected the medical treatment of some patients and even worsened the patient's condition, increasing the risk of hospitalization. In fact, we once again emphasize the importance of long‐term regular treatment for patients with PH. Moreover, it is very necessary to build a treatment system for chronically ill patients in the event of a catastrophe.

## AUTHOR CONTRIBUTIONS

*Concept and design*: Yuqin Chen and Bihua Zhong. *Acquisition, analysis, or interpretation of data*: All authors. *Drafting of the manuscript*: Yuqin Chen and Bihua Zhong. *Critical revision of the manuscript for important intellectual content*: All authors. *Statistical analysis*: All authors. *Administrative, technical, or material support*: Jian Wang and Chunli Liu. All authors contributed to data interpretation, manuscript writing, and critical analysis of the manuscript; and provided final approval for manuscript submission.

## CONFLICT OF INTEREST

The authors declare no conflict of interest.

## ETHICS STATEMENT

The study was approved by the Ethics Committee of the First Affiliated Hospital of Guangzhou Medical University.

## Data Availability

Yuqin Chen and Bihua Zhong had full access to all the data in the study. They took responsibility for the integrity of the data and the accuracy of the data analysis.
